# Case Report: Hydroxychloroquine in an infant with *NKX2-1*-associated interstitial lung disease

**DOI:** 10.3389/fped.2025.1619722

**Published:** 2025-10-16

**Authors:** Di Qing, Xuehua Xu, Tingting Shi, Huifeng Fan, Dongwei Zhang, Diyuan Yang, Gen Lu

**Affiliations:** Department of Respiration, Guangzhou Women and Children’s Medical Center, Guangzhou Medical University, Guangzhou, Guangdong, China

**Keywords:** interstitial lung disease, *NKX2-1* gene, brain–lung–thyroid syndrome, hydroxychloroquine, infant

## Abstract

This study presents a case of brain–lung–thyroid syndrome caused by a pathogenic variant in the *NKX2-1* gene, which is characterized by interstitial lung disease. A 7-month-old female infant was hospitalized for over half a month for cyanosis. The full-term infant developed respiratory distress syndrome soon after delivery, requiring mechanical ventilation, and was diagnosed with congenital hypothyroidism. In the first seven months of life, the infant also showed hypotonia, feeding difficulties, and developmental delays. Chest CT findings demonstrated generalized ground-glass opacities in both lung fields. A heterozygous pathogenic variant of the *NKX2-1* gene [NM_001079668.3:c.583C>T (p.Arg195Trp)] was identified by whole-exome sequencing. The infant received a combination therapy, comprising supplementary thyroxine, nutritional support, high-flow nasal cannula oxygen therapy, and exploratory treatment with hydroxychloroquine. High-flow nasal cannula oxygen therapy was administered after discharge. The patient was followed up for over 2 months, and the patient had changed to low-flow oxygen therapy, although she developed radiographic progression. Studies on hydroxychloroquine for the treatment of interstitial lung diseases are limited. This article describes a case of interstitial lung disease caused by a pathogenic variant in the *NKX2-1* gene, whose oxygen demand decreased after treatment with hydroxychloroquine.

## Introduction

Interstitial lung disease (ILD) in children comprises a large heterogeneous group of respiratory disorders (1). The etiology of interstitial lung disease in children is multitudinous, and with the development of next-generation sequencing methods, it is increasingly being recognized that a growing part of the etiologic spectrum of interstitial lung disease in children is attributed to underlying genetic causes (2, 3). Variants of the *NKX2-1* gene have been associated with “brain–lung–thyroid syndrome” (4). *NKX2-1* encodes thyroid transcription factor 1 (TTF1), which is required for lung development and the expression of surfactant proteins. TTF1 is also expressed in the brain and the thyroid glands. This rare syndrome is characterized by varying degrees of respiratory dysfunction, congenital hypothyroidism, and neurological abnormalities (5). The triad is inconsistent with the heterogeneity of the organs involved and clinical presentation. A variety of phenotypes have been described, including the typical triads, or the presence of any two, or even just one symptom, such as thyroid, pulmonary or neurological symptoms. Pulmonary diseases include the respiratory distress syndrome (RDS) in neonates, interstitial lung disease in young children, and pulmonary fibrosis in older individuals (6–9). In this study, we report the case of a full-term infant with brain–lung–thyroid syndrome and ILD caused by a heterozygous pathogenic variant of *NKX2-1*.

## Case presentation

Our patient was a 7-month-old female infant, who was admitted to the pediatric intensive care unit (PICU) of our hospital with cyanosis for more than half a month. The patient was born full-term by an uncomplicated cesarean section with a birth weight of 3,750 g. Her parents were non-consanguineous with no history of adverse pregnancy. Two hours after delivery, she experienced shortness of breath, developed respiratory distress, and required hospitalization in the neonatal intensive care unit (NICU). During her stay in the NICU, she received 320 mg of intratracheal exogenous surfactant and conventional mechanical ventilation for 10 days. In addition to the respiratory problem, the patient had feeding intolerance and diagnosed with congenital hypothyroidism [thyroid-stimulating hormone (TSH) 58.032 μIU/mL], and hence, she was treated with nasogastric tube feeds and thyroid hormone replacement therapy (8 U/kg). Echocardiography in the NICU revealed patent ductus arteriosus (PDA) and moderate to severe pulmonary hypertension. She was discharged after 24 days of hospitalization, but she still exhibited feeding dysfunction, hypotonia, and neurodevelopmental delay, achieved head control only by the age of 6 months, and remained unable to sit independently until hospitalization. Because of recurrent respiratory infections and repeated shortness of breath and cough, she required several local hospitalizations during the first 7 months.

**Figure 1:**
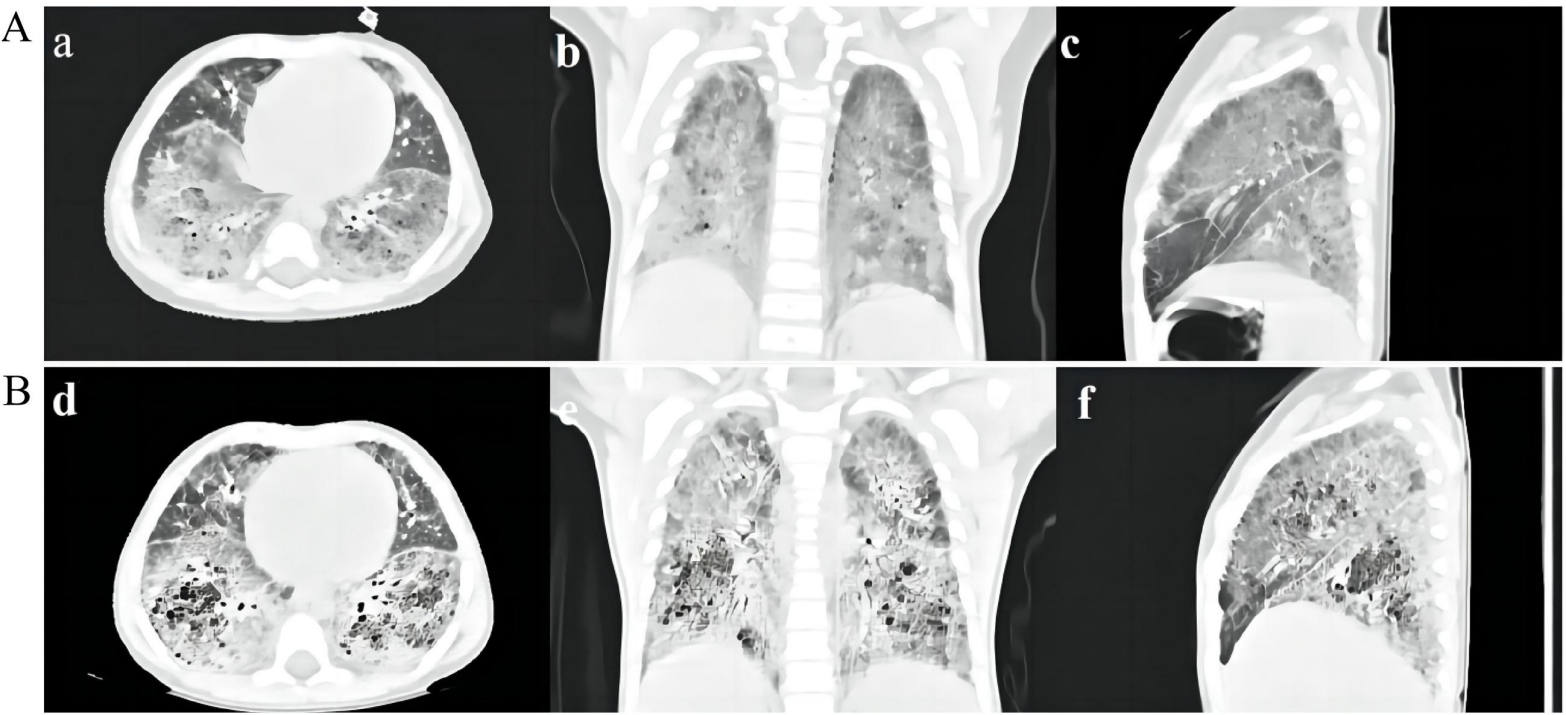
**(A)** HRCT scan of the patient. HRCT scan at 7 months of age showing diffuse reticular and bilateral asymmetrical ground-glass opacities **(a–c)**. **(B)** HRCT scan 2 months after discharge showing multiple cysts and crazy-paving stone signs in both lung fields **(d–f)**.

**Figure 2:**
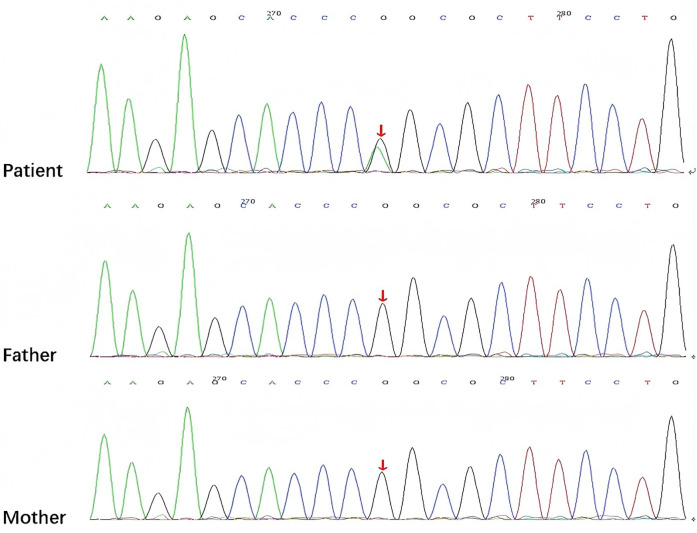
Sanger sequencing showing the *NKX2-1* pathogenic variant [NM_001079668.3:c.583C>T (p.Arg195Trp)] in the patient and her parents detected by exome sequencing (arrow).

**Figure 3:**
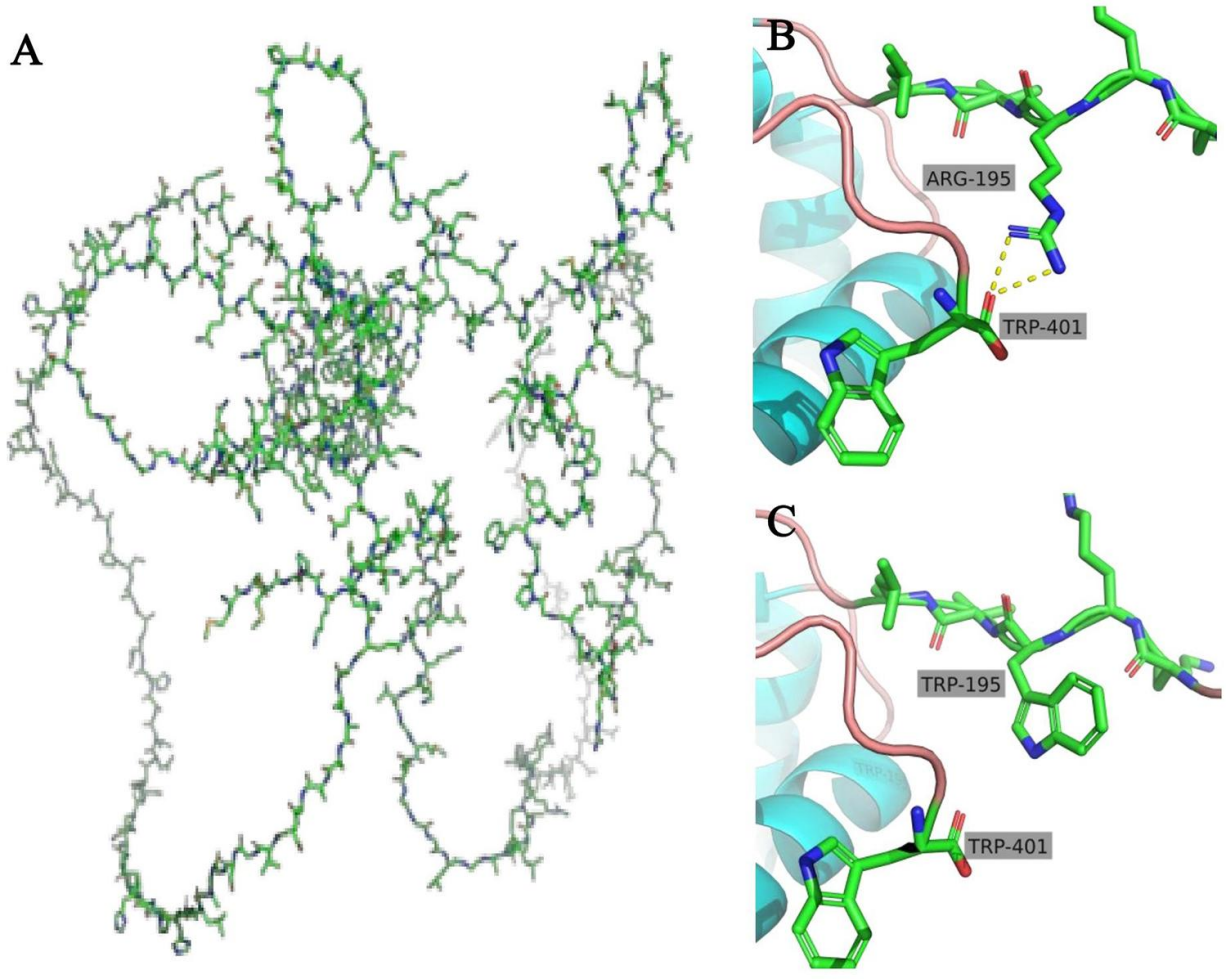
Overall structure of TTF1 **(A)** in the wild type, there are two hydrogen bonds between the 195 amino acid (Arg) and the 401 amino acid (Trp) **(B)**, but when the 195 amino acid (Arg) was substituted with Trp, the hydrogen bonds disappeared **(C)**.

After being admitted to our hospital, her serum thyroid hormones level indicated a significant increase in thyroid-stimulating hormone TSH (63.903 μIU/mL, normal range, NR 0.36–7.63) and low thyroxine levels (10.48 pmol/L, NR 13.17–22.33). Complete blood counts, serum electrolyte levels, liver and kidney function, and myocardial enzyme levels were within the normal ranges. No immune function abnormalities were found. The results of nasopharyngeal swab tests, bronchoalveolar lavage for a respiratory viral panel by polymerase chain reaction (PCR), and blood culture were all negative. Fiberoptic bronchoscopy and alveolar lavage were performed and the bronchoalveolar lavage fluid (BALF) was limpid. Chest radiography revealed multifocal opacities in both lung fields, and chest high-resolution computed tomography (HRCT) revealed diffuse reticular and bilateral patchy ground-glass opacities (Figures 1A-a–c). Owing to hypotonia and neurodevelopmental delay, a brain MRI was performed, the results of which were normal. Echocardiography revealed resolved pulmonary hypertension. Sanger sequencing was performed on the patient and her parents, which identified a heterozygous *de novo* missense pathogenic variant in the *NKX2-1* gene [NM_001079668.3:c.583C>T (p.Arg195Trp)], c.583C>T in exon 3 in the patient; neither of her parents had the pathogenic variant (Figure 2). This missense variant resulted in the substitution of an arginine (Arg) residue with tryptophan (Trp) at codon 195 (p.Arg195Trp). It did not change the secondary structure of the protein but resulted in the disappearance of two hydrogen bonds between amino acids 195 and 401, thus resulting in a dysfunction of the DNA-binding domain (DBD) (Figure 3).

According to the clinical phenotypes and genetic analysis, the patient was confirmed to have brain–lung–thyroid syndrome stemming from a pathogenic variant of the *NKX2-1* gene. Apart from continued administration of thyroid hormone replacement therapy, the infant was treated with nutritional supplementation and high-flow nasal cannula (HFNC) oxygen therapy, in which the parameters showed that the fraction of inspired oxygen (FiO_2_) was 36% and the oxygen flow rate was 20 L/min (the weight of the patient was 6.2 kg). She was suggested to receive glucocorticoid treatment, but in consideration of her young age, poor nutritional status, and adverse reactions to glucocorticoids, her parents refused the therapeutic regimen. Subsequently, she received trial treatment with hydroxychloroquine (HCQ) 10 mg/kg/day. She still required HFNC (FiO_2_, 32%; oxygen flow rate, 12 L/min) and nasogastric feeding upon discharge from the hospital. Following discharge after 2 months, she was off HFNC and required a nasal oxygen supplementation (low-flow oxygen therapy, FiO_2_25%–28% at 0.5–1 L/min), HRCT showed a worsening status that demonstrated a crazy-paving stone sign and multiple cysts (Figures 1B-e,f). HCQ was discontinued following liver insufficiency, and she is still under follow-up.

## Discussion

In our report of a case of ILD caused by a pathogenic variant of the *NKX2-1* gene, the patient's clinical symptoms improved after HCQ treatment. We hope that this report will draw attention to ILD and the treatment of ILD with HCQ. ILD is a group of numerous etiological respiratory disorders, and pathogenic variants in genes encoding surfactant components have been recognized as a growing etiology (10). Currently, known genetic abnormalities associated with interstitial lung disease in children are being identified in the surfactant genes—*SFTPA1*, *SFTPA2*, *SFTPB*, *SFTPC*, *ABCA3*, and *NKX2-1* (2). *NKX2-1* is a homologous transcription factor of the *NKX2* gene family, which is located on chromosome 14, is composed of three exons and two introns, and encodes a nuclear protein named TTF1 with a relative molecular mass of 38,000 (11). In our study, we found that the patient carried a *de novo* NKX2.1 missense mutation (c.583C>T; p.Arg195Trp). Structural modeling revealed a disruption of hydrogen bonds between amino acid 195 and 401, thereby impairing the DBD and transcriptional activity of TTF1 (14, 15). Furthermore, we classified this variant according to the American College of Medical Genetics and Genomics (ACMG) and ClinGen guidelines. Supporting evidence included the following: confirmed *de novo* occurrence (PS2), absence from population databases (PM2), location in a critical functional domain (PM1), assumed *de novo* status without confirmed paternity/maternity testing (PM6), deleterious in silico prediction (PP3), and a phenotype highly specific for NKX2.1-related disorders, namely, the classical brain–lung–thyroid triad (PP4). Based on these criteria, the c.583C>T (p.Arg195Trp) variant is classified as likely pathogenic, further supporting its causal relationship with the clinical presentation observed in our patient. TTF1 plays a vital role in the development and maturation of the thyroid, lung, and central nervous system (14). In the lungs, TTF1 is essential for the expression of surfactant proteins and *ABCA3* genes, thus influencing lung morphology, pulmonary epithelial cells, and their functions, particularly in regulating the diversity of surfactant protein genes and development, including surfactant metabolism and homeostasis (15).

*NKX2-1* gene pathogenic variant–associated clinical phenotypes are highly multitudinous (16); only approximately half of the patients manifest the full triad of brain–lung–thyroid syndrome, approximately a quarter of them show an isolated respiratory phenotype, and one-fifth of them have neurological symptoms associated with the respiratory phenotype (17, 18). In our study, we found that the patient demonstrated typical triad manifestations similar to those found in several previous reports. Our patient had RDS during her neonatal period; at the same time, congenital hypothyroidism was identified. The infant gradually developed neurological symptoms, including hypotonia and feeding difficulties, and subsequently developed ILD. Her imaging features showed representative ground-glass opacities, as previously reported (19), and subsequent HRCT demonstrated the evolution of crazy-paving stone signs and multiple cysts over time. Thus far, no specific therapeutic strategy has been developed for children with *NKX2-1* pathogenic variants (1). General measures are essential, including exogenous surfactant therapy for patients presenting with acute respiratory distress in the neonatal period, oxygen supplementation for chronic hypoxemia, thyroid hormone replacement therapy, and maintenance of nutrition. In addition to general measures, pharmacological therapies such as corticosteroids, HCQ, and azithromycin may play anti-inflammatory and immunosuppressive roles and have been reported to be effective in some cases with *NKX2-1* pathogenic variants (20). Lung transplantation may be the ultimate therapy for prolonging survival in children with end-stage progressive disease (21).

In our case, apart from the general treatment, the patient also received HCQ for 2 months. She showed some clinical improvement in oxygen demand after HCQ treatment despite radiological worsening. This apparent discordance between the improved oxygen demand and the radiological deterioration should be viewed with caution. In pediatric ILD, especially those caused by rare genetic pathogenic variants, clinical and radiological responses may not evolve synchronously over a short observation period. In our patient, the reduction in the oxygen requirement was a tangible and meaningful clinical improvement for daily care, although HRCT changes indicated progression. Such discordance does not necessarily negate the potential effect of HCQ but rather reflects the complexity of disease dynamics and the limitations of short-term assessment. We believe that this case provides valuable insights, suggesting that HCQ may offer transient symptomatic benefits, while highlighting the importance of long-term follow-up and a comprehensive evaluation of *NKX2-1*-associated ILD.

HCQ, which is part of group 4-aminoquinolines, is suitable for not only malaria and connective tissue disease, but also interstitial lung disease (22). Studies on the efficacy of HCQ in the interstitial lung have shown different results for different types of genetic abnormalities. The results of a recent randomized controlled phase 2 trial of HCQ in 35 patients with various forms of interstitial lung disease (caused by the pathogenic variants of *SFTPC*, *ABCA3*, *NKX2.1*, *TBX4*, *COPA*, etc.) did not identify an overall HCQ treatment effect (23). However, case reports and *in vitro* experiments have provided evidence of the efficacy of HCQ in patients carrying *SFTPC* or *ABCA3* variants (12, 24, 25). The exact mechanism of action of HCQ in interstitial lung disease is unknown; however, it may reduce interstitial inflammation and alter intracellular metabolism. HCQ remains controversial in the treatment of lung disease because of the presence of *NKX2-1* pathogenic variants. A retrospective study including 16 patients with variants in *NKX2-1* showed that all symptomatic interstitial lung disease patients benefited from a treatment consisting of steroids, azithromycin, and/or HCQ, but there was little evidence of the effectiveness of HCQ because no patient received single-drug therapy of HCQ, and there was no difference between different combinations of two or three drugs (12).

In accordance with previous reports, it is important to note that hydroxychloroquine exerts pleiotropic effects, including immunomodulatory actions, inhibition of toll-like receptor (TLR) signaling, lysosomal activity, and autophagy. However, its clinical efficacy as a monotherapy for ILD is limited and inconsistent. In clinical practice, particularly in rheumatology and pediatric ILD, hydroxychloroquine is often used in combination with corticosteroids, azathioprine, or mycophenolate mofetil, especially in patients with severe parenchymal involvement, because monotherapy alone rarely achieves sustained improvement. However, in our case, although our patient did not show improved results on imaging, her clinical symptoms alleviated and she showed improvement in terms of the level of oxygen therapy after a single-drug HCQ therapy. We speculate that HCQ may be conducive to the improvement of breathing difficulties in patients with *NKX2-1* pathogenic variants. However, possibly owing to a shorter follow-up period or singularity of the case, we could not confirm the efficacy of HCQ. Furthermore, more randomized or controlled studies with more clinical phenotypes and more combination treatment options are required to evaluate the effects of HCQ treatment on lung disease caused by *NKX2-1* pathogenic variants.

## Limitations and perspectives

Certain inherent limitations of this report are worthy of mention. This report constitutes a single-case observation with a relatively short follow-up period. Nevertheless, even within two months of therapy, our patient demonstrated a measurable reduction in the oxygen demand, which is clinically meaningful in the daily management of severe pediatric ILD. The discordance between clinical and radiological outcomes highlights the complexity of disease evolution in *NKX2-1*-related ILD and suggests that therapeutic responses may vary across different domains. Importantly, our findings underscore the need for long-term follow-up and systematic studies to clarify the role of HCQ, either as a monotherapy or in combination regimens, in this rare genetic context. Rather than diminishing the significance of this case, these limitations provide valuable insights and generate hypotheses for future research on *NKX2-1*-associated ILD.

## Conclusion

When evaluating a full-term neonate with unexplained respiratory distress, and later when the neonate attains childhood with signs and symptoms of interstitial lung disease accompanied by hypothyroidism and neurologic abnormalities, brain–lung–thyroid syndrome associated with the *NKX2-1* pathogenic variant should be considered. HCQ is a treatment measure. The question whether interstitial lung disease in patients with pathogenic variants of *NKX2-1* benefits from HCQ requires further research.

## Data Availability

The data analyzed in this study is subject to the following licenses/restrictions: The datasets for this article are not publicly available due to concerns regarding participant/patient anonymity. Requests to access these datasets should be directed to the corresponding authors.
